# A cross-sectional study of how high-frequency hearing loss impacts cognitive functions in middle-aged-to-older adults

**DOI:** 10.3389/fnagi.2025.1560307

**Published:** 2025-04-28

**Authors:** Dona M. P. Jayakody, Paul McIlhiney, Inge Stegeman, Robert H. Eikelboom

**Affiliations:** ^1^Ear Science Institute Australia, Subiaco, WA, Australia; ^2^Medical School, The University of Western Australia, Crawley, WA, Australia; ^3^Curtin Medical School, Curtin University, Bentley, WA, Australia; ^4^WA Centre for Health and Ageing, The University of Western Australia, Crawley, WA, Australia; ^5^Department of Otorhinolaryngology and Head and Neck Surgery, University Medical Center, Utrecht, Netherlands; ^6^Brain Center, Rudolf Magnus University Medical Center, Utrecht, Netherlands; ^7^Department of Speech-Language Pathology and Audiology, University of Pretoria, Pretoria, South Africa

**Keywords:** hearing loss, cognition, high frequency, working memory, executive function

## Abstract

**Purpose:**

Research on the association between hearing loss and cognition has primarily focused on speech-range hearing frequencies (i.e., 0.5–4 kHz), as these frequencies are most relevant to everyday functioning. However, age-related hearing loss (ARHL) tends to impact higher-frequency hearing first, and more severely. Despite this, limited research has investigated the relationship between high-frequency (i.e., >4 kHz) hearing loss and cognitive impairment. In the current study, we aimed to assess whether high-frequency hearing loss predicts non-verbal cognitive functions (i.e., visuospatial executive function, learning, and memory tasks) above and beyond speech-frequency hearing loss.

**Materials and methods:**

Participants were 241 English-speaking adults, aged 40–88 years, with hearing loss. Audiometrically assessed better-ear, speech-frequency (0.5, 1, 2 & 4 kHz; BE4PTA) and high-frequency (6 & 8 kHz; BE2PTA) hearing loss were compared to cognitive functions measured using non-verbal tests from the Cambridge Neuropsychological Test Automated Battery; covariates included hearing-loss asymmetry, age, sex, premorbid IQ, and mental health measured with the short-form Depression Anxiety Stress Scales.

**Results:**

While correlation analyses demonstrated that all measured cognitive faculties were associated with both BE4PTA and BE2PTA, hierarchical linear regression analyses demonstrated that only BE4PTA predicted cognitive flexibility and working-memory ability after controlling for covariates; age primarily accounted for BE2PTA’s cognitive effects.

**Conclusion:**

While both speech and higher-frequency hearing loss were associated with poorer cognition, only the former demonstrated effects beyond those of ageing. However, the present study only investigated two frequencies in the higher range, encouraging broader investigation of higher-frequency hearing’s cognitive effects in the future.

## 1 Introduction

Hearing loss and cognitive impairment are both highly prevalent, with each contributing to significant detriment in society (Haile et al., 2021; Nichols et al., 2022). Furthermore, an association between hearing loss and cognitive impairment has been reliably demonstrated (Livingston et al., 2020; Powell et al., 2022; Sher and Owens, 1974; Taljaard et al., 2016). However, while hearing loss typically has a larger impact on higher hearing frequencies (i.e., > 4 kHz; Cruickshanks et al., 1998; Davis et al., 2016), most studies investigating the relationship between hearing loss and cognition have focused on lower speech-related frequencies (i.e., 0.5–4 kHz; Bucks et al., 2016; Jayakody et al., 2018; Lin et al., 2011). Thus, literature on the relationship between high-frequency hearing loss and cognition is lacking; a paucity that the current paper aimed to address.

Broadly, several studies have hitherto demonstrated that hearing loss in the speech frequencies (i.e., 0.5–4 kHz) is related to cognitive functioning (Powell et al., 2022; Jayakody et al., 2018; Meister et al., 2016). Jayakody et al. (2018) investigated whether speech-frequency hearing loss predicted non-verbal cognitive function in a sample of Australian adults and older adults. Results showed that greater speech-frequency hearing loss predicted poorer performance on visuospatial-working-memory and paired-associates-learning tasks. Meanwhile, Meister et al. (2016) investigated how hearing loss related to working-memory capacity and speech recognition. Results demonstrated that those with hearing loss incurred greater demands on their working-memory capacity during speech recognition, suggesting that hearing loss impacts cognitive ability negatively by placing greater load on cognitive resources. Therefore, past literature has provided evidence that speech-frequency hearing loss and cognition correlate to some degree.

However, hearing loss tends to affect high-frequency hearing more severely than lower-frequency hearing. Using a sample of older adults aged 70 years and above, Lin et al. (2011) showed that prevalence of hearing loss in higher frequencies (i.e., 3, 4, 6, & 8 kHz) was 90.9% in the better ear and 95.2% in the poorer ear; these estimates exceeded those of speech-frequency hearing loss, which were 63.1 and 75.1%, respectively. Furthermore, although the majority of speech sounds occur in the 0.5 to 4 kHz range, many consonants have spectral energy in the higher frequency ranges. For example, in English, the spectral peaks of the letters “s,” “z,” and “v” are 4 to 4.5 kHz, 4 to 7 kHz, and close to 8 kHz, respectively (Reetz and Jongman, 2011)—similar observations have been made for Arabic and French (Al-Khairy, 2005; de Manrique and Massone, 1981). Thus, an inability to understand high-frequency sounds could hinder one’s ability to understand speech, especially in demanding listening situations (Festen and Plomp, 1990; Summers and Molis, 2004).

Considering the above, if high-frequency hearing loss tends to be more severe than speech-frequency hearing loss (Cruickshanks et al., 1998; Davis et al., 2016) and potentially impacts everyday functioning (Festen and Plomp, 1990; Summers and Molis, 2004), then it could impact cognitive functioning in a similar manner to speech-frequency hearing loss (Jayakody et al., 2018; Meister et al., 2016). However, research on the relationship between high-frequency hearing loss and cognition has been limited (Diao et al., 2021; Waechter et al., 2022; Fu et al., 2023; Kestens et al., 2021). Diao et al. (2021) tested whether Chinese older adults (≥ 60 years) with normal high-frequency hearing (4 kHz, 8 kHz; ≤25 dB) or high-frequency hearing loss (4 kHz, 8 kHz; >25 dB) differed on scores from the *Montreal Cognitive Assessment* (MoCA; Nasreddine et al., 2005). Results showed that, although overall MoCA scores did not differ between groups, the language and abstract scores were significantly worse in the high-frequency-hearing-loss group. Also testing a Chinese older-adult sample (aged ≥ 60 years), Fu et al. (2023) showed that worsening high-frequency hearing loss (4 kHz, 6 kHz, and 8 kHz) correlated with worsening scores on the hearing-impaired version of the MoCA (HI-MoCA; Lin et al., 2017) over a 12-month period. Meanwhile, Waechter et al. (2022), testing Swedish university students aged 23 to 66 years, found that extended high-frequency hearing loss (i.e., 10–16 kHz) was significantly negatively associated with performance on a cognitive task designed to simulate real-life office work (for task details, see Hua et al., 2014). Finally, Kestens et al. (2021) investigation with Belgian younger-to-older adults (aged 16 to 69 years) showed that lower hearing sensitivity coincided with slower verbal processing speed. However, note that hearing in the study by Kestens and colleagues was measured as a composite of all octave frequencies from 0.5 to 8 kHz, meaning that high-frequency hearing was not isolated. Furthermore, the sample size for the Diao et al. (2021) and Waechter et al. (2022) studies was small (*N* = 58 and *N* = 76, respectively); moreover, the MoCA used by Diao and colleagues is only a brief cognitive screener, and the office-like task used by Waechter and colleagues, while naturalistic, is not an in-depth measure of cognitive ability. Meanwhile, the results of Fu et al. (2023) are difficult to generalise to populations speaking non-tonal languages. Thus, there is need for an investigation of the association between high-frequency hearing loss and cognition using more robust cognitive measures.

Therefore, in the current study, we aimed to investigate whether high-frequency hearing loss (i.e., 6 and 8 kHz) impacts cognition in middle-aged-to-older adults beyond the influence of speech-frequency hearing loss. Hearing loss was measured with pure-tone audiometry. Following precedent (Jayakody et al., 2018), cognition was measured using objective, nonverbal tests from the *Cambridge Neuropsychological Test Automated Battery* that were sensitive to MCI and Alzheimer’s (Égerházi et al., 2007); non-verbal tests were used to reduce the confounding effect of participants’ hearing loss on their test performance. To control for other potential confounding effects, demographic, hearing-asymmetry, premorbid intelligence, and mental-health variables were included as covariates.

It was firstly hypothesised that greater speech and high-frequency hearing loss would predict lower cognitive ability. Accordingly, we predicted our cognitive measures to significantly correlate with speech and high-frequency hearing-loss estimates. It was further hypothesised that greater high-frequency hearing loss would predict lower cognitive functioning beyond the influence of speech-frequency hearing loss. As such, we predicted significant standardised *β*’s between high-frequency hearing-loss estimates and cognitive outcomes.

## 2 Materials and methods

In this individual-differences study, we used a cross-sectional design with two independent variables (speech-frequency and high-frequency hearing loss), five covariates (hearing-loss, speech and high frequency hearing asymmetry, age, premorbid IQ, sex), and one dependent variable (cognitive function). Four subdimensions of cognition were measured (cognitive flexibility, short-term visual recognition memory, visuospatial episodic memory & learning, visuospatial working memory).

### 2.1 Participants

A total of 284 Australian, English-speaking participants who met inclusion criteria were recruited through community advertisement, and from the Ear Science Institute Australia hearing clinics; however, after exclusions, 241 participants remained, with 115 males and 126 females who had an average age of *M* = 64.77 (*SD* = 11.35, Range = 40–88) years. Inclusion criteria comprised those who were at least 40 years old, in a general state of good health, not in a dependent relationship (e.g., under caregiver supervision), and not restricted from performing required tasks due to a history of an underlying physical, medical, or mental conditions. Participants were excluded listwise (after inclusion criteria were met) from analysis for having missing data from any of the demographic, auditory, or cognitive measures; note that results were similar if pairwise exclusion was adopted instead, as reported in the Online Supplement (https://osf.io/pg5fm/).

### 2.2 Materials

#### 2.2.1 Audiometry

Following otoscopic assessment, bilateral air-conduction thresholds for 0.5, 1, 2, 4, 6, and 8 kHz were obtained through standard audiometric assessment; these were conducted by a qualified audiologist in a soundproof booth using a Kuduwave Pro extended-high-frequency diagnostic audiometer (eMOYO, Johannesburg, South Africa). For analyses, 0.5, 1, 2, and 4 kHz thresholds in the better ear represented speech-frequency hearing (BE4PTA), while 6 and 8 kHz thresholds in the better ear represented high-frequency hearing (BE2PTA). If hearing loss was detected and otoscopic assessment indicated potential middle-ear pathology, then bone-conduction thresholds were obtained; if bone-conduction thresholds also demonstrated hearing loss, then tympanometry was conducted for confirmation. Those with middle-ear pathologies, or any other non-age-related hearing losses, were included in analyses, with supplementary analyses suggesting that this had no effect on our results (see Online Supplement) (https://osf.io/pg5fm/). We also included in analyses those with asymmetrical hearing loss (i.e., a greater degree of loss in one ear) in the speech-frequency (4PTAsym) or high-frequency (2PTAsym) ranges; the influence of this was controlled for by including, as a covariate, the absolute difference in hearing thresholds between ears.

#### 2.2.2 Premorbid IQ measure

Prior to cognitive assessment, all participants completed the Revised National Adult Reading Test (NART-R) to assess premorbid IQ (Nelson and Willison, 1991). The task involved participants reading aloud a list of 50 irregularly spelled English words, one at a time, while the researcher assessed the correctness of pronunciation and records errors. The number of errors were then used to calculate an IQ estimate.

#### 2.2.3 Mental-health assessment

Mental health was assessed with the short-form Depression Anxiety Stress Scale (DASS-21; Lovibond and Lovibond, 1995). This assessment measures the subjective severity and frequency of depression, anxiety, and stress symptoms over the previous 7 days. The depression, anxiety, and stress subdimensions each use seven items, with each item presenting a statement (e.g., “I felt that I was using a lot of nervous energy”) that is responded to on a four-point Likert scale (i.e., *Never* [0], *Sometimes* [1], *Often* [2], *Almost Always* [3]). Item Likert ratings for each subdimension are summed and multiplied by 2 to provide depression, anxiety, and stress composite scores; these scores range from 0 to 42, with higher scores indicating more severe and frequent symptoms.

#### 2.2.4 Cognitive assessment

Cognitive ability was assessed using the Cambridge Neuropsychological Test Automated Battery (CANTAB; Cambridge Cognition Ltd., UK), installed on a computer with an integrated touchscreen (Dell, Inspiron One, with Windows 8.1 platform). Participants completed the following modules of the CANTAB, which were selected for their demonstrated sensitivity to mild cognitive impairment or Alzheimer’s disease (Égerházi et al., 2007), and use of non-verbal stimuli that help avoid confounding effects from hearing loss: Attention Switching Task (AST), Delayed Matching to Sample (DMS), Paired Associates Learning (PAL), and Spatial Working Memory (SWM). Further, the Motor Screening Task (MOT) was used to screen for participants who were unable to follow visual instructions or use a touchscreen computer. Cognitive testing was conducted in a sound-treated room and lasted approximately 90 min. To avoid fatigue, participants were given breaks between test modules. All participants provided written consent prior to taking part in the study, and ethics approval was provided by The University of Western Australia’s Human Research Ethics Committee (RA/4/1/7368).

##### 2.2.4.1 Attention switching task (AST)

This task is a measure of cognitive flexibility (i.e., set shifting), which is a top-down, executive process (see Miyake et al., 2000). In each trial, an arrow appeared on the right or left of the screen, pointing either to the left or right; a cue at the top of the screen then stated “Direction” or “Side,” instructing participants either to indicate the direction the arrow is pointing, or the side of the screen the arrow is on. Responses were recorded using “Left” and “Right” touchscreen buttons. Performance was quantified by the total percentage of correct trials.

##### 2.2.4.2 Delayed matching to sample (DMS)

This task assesses both simultaneous visual matching and short-term visual memory (Sharma, 2021). For each trial, participants were shown a complex visual pattern (sample) in the top middle of the screen; after a potential brief delay (0, 4, or 12 s), four other complex patterns were then shown at the bottom of the screen. Participants had to select which of the four other patterns matched the sample pattern, namely by touching the correct pattern with the touchscreen. Performance was measured with the total percentage of correct trials.

##### 2.2.4.3 Paired associates learning (PAL)

This task measures participants’ visuospatial episodic memory and learning skills through a pattern-matching task (Sharma, 2021). Each trial presented six white boxes on the screen, which each briefly, and sequentially, revealed patterns of differing shape and colour. Subsequently, patterns were revealed in the centre of the screen, and participants had to match these patterns to one of the six white boxes that they previously appeared in. Participants responded by tapping on the correct box with the touchscreen. Performance was measured by total number of errors, multiplied by −1 to facilitate ease of interpretation (i.e., make higher scores represent better cognition).

##### 2.2.4.4 Spatial working memory (SWM)

This task assesses participants’ ability to manipulate spatial information in working memory, including the use of heuristic memory strategies (Sharma, 2021); the former is considered to be an executive function (see Miyake et al., 2000). Each trial presents a number of boxes, with one box containing a token per trial; a trial can contain 3, 4, 6 or 8 boxes, representing increasing difficulty. Searching the same box within a trial represents a within search error, while searching a box that held the token in a previous trial is a between search error. A strategy score is also used on the more-difficult trials (i.e., 6 & 8 boxes), which tracks the use of predetermined search sequences (i.e., starting each trial with the same box). Responses were recorded by tapping boxes on the touchscreen. Performance was quantified by the total number of errors (within + between) and the strategy scores, which were multiplied by −1 to facilitate easier interpretation.

### 2.3 Procedure

Following the provision of written informed consent, participants were shown to a sound-proof booth for audiometric testing. Subsequently, participants completed the NART-R before being provided with the touchscreen computer to complete the CANTAB. Test order was the same for all participants, and total testing time was approximately 120 min.

### 2.4 Statistical analysis

All analyses were performed in SPSS v29 (IBM Corp, 2023). Descriptive analyses were firstly performed to assess the normality and homoscedasticity of our data variables. Correlational analyses were then used to identify the variables suitable for use in our hierarchical multiple-regression analyses; that is, the variables that demonstrated significant correlations. Subsequently, we conducted hierarchical multiple-regression analyses to ascertain whether high-frequency hearing loss (BE2PTA) influenced cognition above and beyond the influence of speech-frequency hearing loss (BE4PTA) and the covariates of BE4PTA asymmetry (4PTAsym), BE2PTA asymmetry (2PTAsym), age, premorbid IQ (NART-R), sex, and mental health (i.e., subjective depression, anxiety, & stress symptoms). In these regression analyses, we entered BE4PTA and BE2PTA at step 1, then sequentially entered 4PTAsym and 2PTAsym, age, NART-R, sex, depression, anxiety, and stress at steps 2, 3, 4…8, respectively. A comprehensive report of results has been provided in the Online Supplement (https://osf.io/pg5fm/).

Point estimate 95% *CI*s were calculated using bootstrapping (2,000 samples) with bias correction and acceleration; note that wild bootstrapping was used for regression analyses to control for potential effects of heteroscedasticity. While we used standardised beta-weights for regression analyses, the associated 95% *CI*s were unstandardised. Effect sizes for Pearson’s *r* correlations were based on guidelines from Gignac and Szodorai (2016) (small, 0 < *r* ≤ 0.10; medium 0.10 < *r* ≤ 0.20; large 0.20 < *r* ≤ 0.30).

## 3 Results

### 3.1 Descriptive analyses

**Figure 1:**
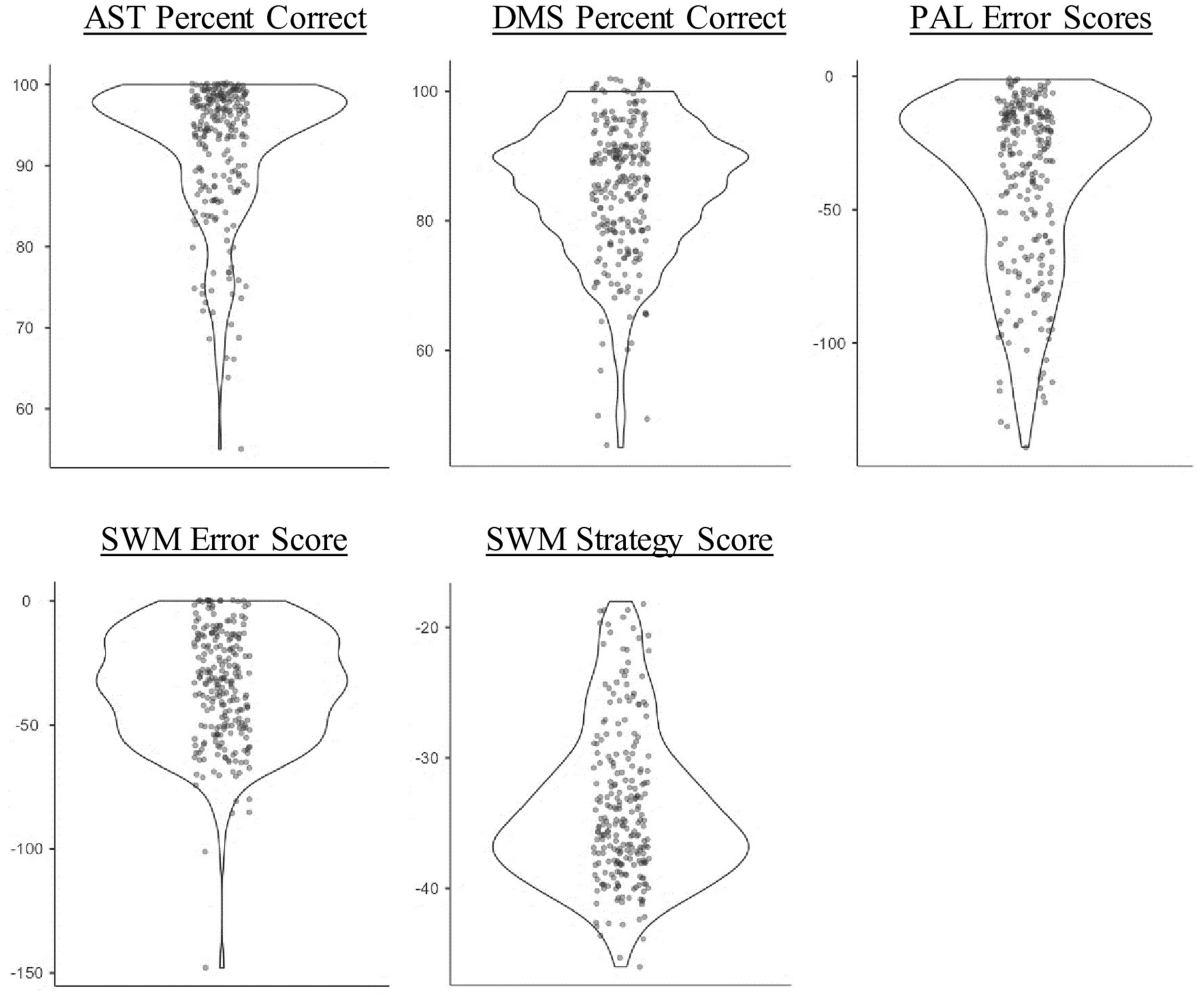
Violin plots showing the data distribution of proportion-correct scores in the attention switch task (AST) and delayed matching-to-sample (DMS) task, error scores in the paired associates learning (PAL) and spatial working memory (SWM) tasks, and strategy scores in the SWM task (*N* = 241). All error scores and the strategy scores have been multiplied by −l, so that higher and lower scores mean better and worse performance, respectively.

As shown in Table 1, all measures fell within the acceptable-normality criteria (skew < |2| & kurtosis < |9|; Schmider et al., 2010; West et al., 1995). However, as demonstrated by the violin plots in Figure 1, there was some perceivable skew in the cognitive measures, with all but the SWM strategy score showing ceiling effects. Note that further descriptive statistics for our data can be found in the Online Supplement (https://osf.io/pg5fm/).

**Table 1:** Descriptive statistics for hearing, cognition, and demographic measures of participants (*N* = 241).

Measure	Mean (*SD*)	Range	Skewness	Kurtosis
BE4PTA_(dB)_	32.08 (21.73)	1.25, 117.50	1.41	2.00
BE2PTA_(dB)_	49.92 (29.52)	−5, 115	0.25	−0.71
4PTAsym_(dB)_	12.36 (20.40)	0, 110	2.41	5.73
2PTAsym_(dB)_	14.03 (17.91)	0, 110	2.23	5.15
Age_(years)_	64.77 (11.35)	40, 88	−0.36	−0.62
NART-R_(IQ)_	112.08 (7.52)	87, 126	−0.73	0.35
AST_(%)_	92.30 (8.43)	55, 100	−1.58	2.28
DMS_(%)_	85.06 (10.31)	45, 100	−0.89	1.12
PAL_(errors)_	−39.56 (32.67)	−139, −1	−0.99	0.02
SWM_(total errors)_	−34.56 (22.52)	−148, 0	−0.71	1.64
SWM_(strategy)_	−33.83 (5.92)	−46, −18	0.74	−0.00
Depression	4.70 (5.29)	0, 32	1.88	4.63
Anxiety	4.43 (4.80)	0, 24	1.39	1.84
Stress	8.90 (6.34)	0, 30	0.64	0.40

dB, Decibels; 4PTAsym, Speech-Frequency Hearing-Loss Asymmetry; 2PTAsym, High-Frequency Hearing-Loss Asymmetry; NART-R, Revised National Adult Reading Test; BE4PTA, Speech Better-Ear Pure-Tone Average; BE2PTA, High-Frequency Better-Ear Pure-Tone Average; AST, Attention-Switching Task; DMS, Delayed Matching to Sample; PAL, Paired Associates Learning; SWM, Spatial Working Memory.

### 3.2 Correlation analyses

As seen in Table 2, our speech-frequency (BE4PTA) and high-frequency (BE2PTA) hearing-loss measures correlated significantly and positively with each other. Hearing-loss-asymmetry measures in the speech-frequency range (4PTAsym) correlated with those in the high-frequency range (2PTAsym) positively with large effect size; although, only 2PTAsym was associated with hearing-loss severity, demonstrating a small negative correlation with BE4PTA. Thus, asymmetry was similar between both hearing-frequency ranges, and generally had very little relation to hearing-loss severity.

**Table 2:** Correlations between predictors (1, 2), covariates (3, 4, 5, 6, 7, 13, 14, 15), and dependent variables (8, 9, 10, 11, 12) with bootstrapped *p*-values (*N* = 241).

Measure	(1)	(2)	(3)	(4)	(5)	(6)	(7)	(8)	(9)	(10)	(11)	(12)	(13)	(14)	(15)
(1) 4PTA	–														
(2) 2PTA	**0.85§**	–													
(3) 4PTAsym	0.02	0.08	–												
(4) 2PTAsym	−0.14*	−0.12	0.**83§**	–											
(5) Age	0.09	**0.25§**	−0.05	−0.09	–										
(6) NART	**−0.34§**	**−0.28§**	−0.02	0.04	0.15*	–									
(7) Sex	−0.11	**−0.19†**	0.01	0.06	**−0.24§**	0.08	–								
(8) AST	**−0.28§**	**−0.28§**	0.02	0.08	**−0.43§**	**0.19†**	0.05	–							
(9) DMS	**−0.20†**	**−0.26§**	−0.11	−0.07	**−0.35§**	**0.20†**	**0.23§**	**0.42§**	–						
(10) PAL	−0.17*	**−0.25§**	0.09	0.10	**−0.39§**	0.12	**0.17†**	**0.42§**	**0.47§**	–					
(11) SWM_T_	**−0.26§**	**−0.26§**	0.10	0.10	**−0.49§**	0.10	0.05	**0.57§**	**0.38§**	**0.52§**	–				
(12) SWM_S_	**−0.24§**	**−0.18†**	0.04	0.05	**−0.33§**	0.06	−0.09	**0.38§**	**0.23§**	**0.27§**	**0.69§**	–			
(13) Dep	**0.24§**	**0.26§**	**0.22§**	0.07	0.04	−0.02	−0.02	−0.01	−0.09	−0.05	−0.02	0.02	–		
(14) Anx	0.14*	0.12	**0.25§**	**0.20†**	0.03	−0.00	−0.01	−0.04	−0.04	−0.01	−0.05	0.00	**0.63§**	–	
(15) Str	0.14*	0.13	**0.21†**	0.13*	**−0.17†**	0.05	0.09	0.08	0.00	0.06	0.05	0.06	**0.59§**	**0.56§**	–

*<0.05; †<0.01; §<0.001. NART-R, Revised National Adult Reading Test; BE4PTA, Speech Better-Ear Pure-Tone Average; BE2PTA, High-Frequency Better-Ear Pure-Tone Average; AST, Attention-Switching Task; DMS, Delayed Matching to Sample; PAL, Paired Associates Learning; SWM, Spatial Working Memory. Bold values indicate statistically significant effects.

Concerning demographic covariates, BE4PTA and BE2PTA, respectively, demonstrated large and medium negative correlations with the NART-R measure, suggesting that premorbid IQ was higher when hearing loss in both frequency ranges was lower. NART-R also had a small positive correlation with age, implying higher premorbid IQ in older participants. Of the hearing measures, only BE2PTA correlated with age (medium & positive) and sex (small and negative; coded as male = 1 & female = 2), intimating that high-frequency hearing loss was generally greater at older ages and in males. Age correlated with all cognitive measures, with older age being associated with worse cognitive performance. NART-R only correlated (weakly and positively) with AST and DMS measures (percent correct), while sex showed moderate and small (positive) correlations with DMS and PAL measures, respectively.

As for mental-health measures, BE4PTA, BE2PTA, and 4PTAsym had small-to-medium positive correlations with depression, anxiety, and stress measures, while 2PTAsym only had a medium positive correlation with anxiety measures. Stress also correlated moderately and negatively with age. Depression, anxiety, and stress showed large positive correlations with each other. Mental-health measures did not correlate with cognitive measures.

Regarding hearing and cognition, both 4PTAsym and 2PTAsym did not correlate with cognitive measures. Moreover, BE4PTA and BE2PTA showed small-to-medium, negative correlations with the AST, DMS, PAL, and SWM measures; thus, greater hearing loss in both frequency ranges was generally associated with worse cognitive performance. Accordingly, for regression analyses, all cognitive measures were used.

### 3.3 Hierarchical multiple regression of hearing loss, cognition, and relevant covariates

Hierarchical multiple-regression analyses were run for each of the five cognitive measures (AST, DMS, PAL, SWM_total,_ SWM_strategy_), including covariates (BE4PTA & BE2PTA asymmetry, age, NART, sex, depression, anxiety, stress) to assess the relative contributions of BE4PTA and BE2PTA to cognitive performance. Please refer to Tables 3–7 to see the results for the AST, DMS, PAL, SWMTE, and SWM_strategy_ models, respectively. Results of step-1 models demonstrated significant negative standardised *β*s for BE2PTA in the DMS and PAL models; this suggested that high-frequency hearing loss accounted for unique variation in visuospatial memory and learning ability that speech-frequency hearing loss did not. Further, BE4PTA demonstrated a significant negative β in the SWM_strategy_ model, suggesting that greater speech-frequency hearing loss uniquely predicted more reliance on rote strategy in the SWM task. Adding hearing-asymmetry measures at step 2 did not change the step-1 results, though the 4PTAsym measure produced a significant positive *β* in the SWMTE model; note that this effect was only significant after bootstrapping, and intimated that, after accounting for hearing-loss severity, greater asymmetry in speech-frequency hearing predicted better spatial working memory.

**Table 3:** Wild bootstrapped model estimates for steps 1 to 8 in hierarchical regression analyses of whether better-ear speech-frequency hearing (BE4PTA) and better-ear high-frequency hearing (BE2PTA) predict performance on the attention switching task, controlling for the covariates of speech-frequency-hearing asymmetry, high-frequency-hearing asymmetry, age, premorbid intelligence (NART-R), sex, depression, anxiety, and stress (*N* = 241).

Model		*B*	*β*	Sig. (two-tailed)	BCa 95% Confidence interval^a^	
					Lower	Upper
1	(Constant)	**96.274**		**<0.001**	**94.767**	**97.858**
	BE4PTA	−0.061	−0.156	0.131	−0.145	0.029
	BE2PTA	−0.041	−0.145	0.124	−0.089	−0.004
2	(Constant)	**95.977**		**<0.001**	**94.175**	**97.726**
	BE4PTA	−0.058	−0.150	0.142	−0.144	0.031
	BE2PTA	−0.042	−0.149	0.138	−0.092	−0.003
	4PTAsym	0.006	0.014	0.878	−0.072	0.088
	2PTAsym	0.013	0.028	0.757	−0.067	0.098
3	(Constant)	**115.226**		**<0.001**	**109.803**	**120.844**
	BE4PTA	**−0.134**	**−0.347**	**0.002**	**−0.219**	**−0.048**
	BE2PTA	0.037	0.129	0.230	−0.017	0.089
	4PTAsym	−0.014	−0.034	0.732	−0.093	0.066
	2PTAsym	0.017	0.036	0.699	−0.062	0.099
	Age	−0.316	−0.426	**<0.001**	**−0.400**	**−0.237**
4	(Constant)	**91.483**		**<0.001**	**75.010**	**108.912**
	BE4PTA	**−0.113**	**−0.290**	**0.009**	**−0.203**	**−0.023**
	BE2PTA	0.041	0.143	0.180	−0.013	0.093
	4PTAsym	−0.011	−0.027	0.778	−0.090	0.068
	2PTAsym	0.013	0.028	0.765	−0.066	0.095
	Age	**−0.344**	**−0.463**	**<0.001**	**−0.428**	**−0.264**
	NART-R	**0.220**	**0.196**	**0.001**	**0.087**	**0.345**
5	(Constant)	**93.678**		**<0.001**	**76.747**	**111.774**
	BE4PTA	**−0.107**	**−0.277**	**0.014**	**−0.197**	**−0.018**
	BE2PTA	0.035	0.122	0.266	−0.019	0.087
	4PTAsym	−0.011	−0.027	0.776	−0.090	0.068
	2PTAsym	0.014	0.030	0.743	−0.064	0.095
	Age	**−0.358**	**−0.482**	**<0.001**	**−0.443**	**−0.278**
	NART-R	**0.230**	**0.205**	**<0.001**	**0.100**	**0.354**
	Sex	−1.514	−0.090	0.103	−3.156	−0.001
6	(Constant)	**94.055**		**<0.001**	**77.351**	**111.891**
	BE4PTA	**−0.110**	**−0.285**	**0.012**	**−0.199**	**−0.022**
	BE2PTA	0.033	0.115	0.303	−0.022	0.086
	4PTAsym	−0.022	−0.053	0.617	−0.103	0.063
	2PTAsym	0.022	0.046	0.627	−0.060	0.106
	Age	**−0.357**	**−0.481**	**<0.001**	**−0.443**	**−0.277**
	NART-R	0.225	0.200	**0.001**	**0.092**	**0.349**
	Sex	−1.536	−0.091	0.097	−3.190	−0.010
	Depression	0.099	0.062	0.410	−0.116	0.336
7	(Constant)	**94.071**		**<0.001**	**77.414**	**111.907**
	BE4PTA	**−0.107**	**−0.275**	**0.018**	**−0.198**	**−0.018**
	BE2PTA	0.030	0.105	0.353	−0.024	0.083
	4PTAsym	−0.023	−0.055	0.606	−0.104	0.062
	2PTAsym	0.027	0.057	0.562	−0.055	0.112
	Age	**−0.355**	**−0.478**	**<0.001**	**−0.441**	**−0.274**
	NART-R	**0.225**	**0.200**	**0.001**	**0.093**	**0.350**
	Sex	−1.553	−0.092	0.094	−3.182	−0.032
	Depression	0.156	0.098	0.246	−0.106	0.419
	Anxiety	−0.099	−0.057	0.599	−0.426	0.234
8	(Constant)	**94.068**		**<0.001**	**77.431**	**111.971**
	BE4PTA	**−0.107**	**−0.275**	**0.019**	**−0.198**	**−0.018**
	BE2PTA	0.030	0.105	0.367	−0.025	0.083
	4PTAsym	−0.023	−0.055	0.609	−0.105	0.062
	2PTAsym	0.027	0.057	0.565	−0.056	0.113
	Age	**−0.355**	**−0.478**	**<0.001**	**−0.442**	**−0.273**
	NART-R	**0.224**	**0.200**	**<0.001**	**0.090**	**0.352**
	Sex	**−1.555**	**−0.092**	0.097	**−3.223**	**−0.011**
	Depression	0.155	0.097	0.244	−0.107	0.417
	Anxiety	−0.101	−0.057	0.636	−0.461	0.261
	Stress	0.003	0.002	0.978	−0.188	0.203

BE4PTA, Speech Better-Ear Pure-Tone Average; BE2PTA, High-Frequency Better-Ear Pure-Tone Average; 4PTAsym, Speech-Frequency Hearing-Loss Asymmetry; 2PTAsym, High-Frequency Hearing-Loss Asymmetry; NART-R, Revised National Adult Reading Test. ^a^Unless otherwise noted, bootstrap results are based on 2,000 wild bootstrap samples. Bold values indicate statistically significant effects.

**Table 4:** Wild bootstrapped model estimates for steps 1 to 8 in hierarchical regression analyses of whether better-ear speech-frequency hearing (BE4PTA) and better-ear high-frequency hearing (BE2PTA) predict performance on the delayed matching-to-sample task, controlling for the covariates of speech-frequency-hearing asymmetry, high-frequency-hearing asymmetry, age, premorbid intelligence (NART-R), sex, depression, anxiety, and stress (*N* = 241).

Model		*B*	*β*	Sig. (two-tailed)	BCa 95% Confidence interval^a^	
					Lower	Upper
1	(Constant)	**89.442**		**<0.001**	**87.501**	**91.344**
	BE4PTA	0.035	0.073	0.472	−0.054	0.109
	BE2PTA	**−0.112**	**−0.321**	**0.003**	**−0.191**	**−0.026**
2	(Constant)	**90.451**		**<0.001**	**88.121**	**92.896**
	BE4PTA	0.029	0.060	0.562	−0.061	0.102
	BE2PTA	**−0.113**	**−0.323**	**0.003**	**−0.193**	**−0.022**
	4PTAsym	0.002	0.003	0.972	−0.104	0.101
	2PTAsym	−0.058	−0.100	0.363	−0.174	0.058
3	(Constant)	**108.381**		**<0.001**	**101.283**	**113.594**
	BE4PTA	−0.043	−0.090	0.395	−0.135	0.034
	BE2PTA	−0.039	−0.111	0.306	−0.116	0.045
	4PTAsym	−0.017	−0.034	0.739	−0.127	0.085
	2PTAsym	−0.055	−0.095	0.392	−0.168	0.058
	Age	**−0.295**	**−0.324**	**<0.001**	**−0.418**	**−0.151**
4	(Constant)	**75.707**		**<0.001**	**54.870**	**94.732**
	BE4PTA	−0.012	−0.026	0.816	−0.105	0.068
	BE2PTA	−0.033	−0.095	0.380	−0.111	0.050
	4PTAsym	−0.013	−0.026	0.789	−0.123	0.088
	2PTAsym	−0.060	−0.104	0.346	−0.174	0.052
	Age	**−0.333**	**−0.366**	**<0.001**	**−0.459**	**−0.190**
	NART-R	**0.303**	**0.221**	**<0.001**	**0.140**	**0.463**
5	(Constant)	**72.076**		**<0.001**	**51.172**	**90.899**
	BE4PTA	−0.021	−0.044	0.695	−0.113	0.057
	BE2PTA	−0.023	−0.067	0.534	−0.102	0.062
	4PTAsym	−0.013	−0.026	0.793	−0.122	0.088
	2PTAsym	−0.062	−0.107	0.331	−0.178	0.052
	Age	**−0.310**	**−0.341**	**<0.001**	**−0.441**	**−0.156**
	NART-R	**0.286**	**0.209**	**<0.001**	**0.122**	**0.447**
	Sex	2.504	0.122	0.055	−0.377	5.794
6	(Constant)	**71.797**		**<0.001**	**50.775**	**90.716**
	BE4PTA	−0.019	−0.039	0.715	−0.111	0.060
	BE2PTA	−0.022	−0.062	0.569	−0.101	0.064
	4PTAsym	−0.005	−0.010	0.924	−0.111	0.102
	2PTAsym	−0.067	−0.117	0.295	−0.184	0.044
	Age	**−0.311**	**−0.342**	**<0.001**	**−0.442**	**−0.158**
	NART-R	**0.290**	**0.212**	**0.001**	**0.126**	**0.454**
	Sex	2.520	0.122	0.054	−0.395	5.819
	Depression	−0.073	−0.038	0.586	−0.320	0.160
7	(Constant)	**71.778**		**<0.001**	**50.731**	**90.767**
	BE4PTA	−0.023	−0.048	0.664	−0.117	0.059
	BE2PTA	−0.018	−0.052	0.630	−0.096	0.070
	4PTAsym	−0.004	−0.008	0.939	−0.110	0.102
	2PTAsym	−0.073	−0.127	0.245	−0.189	0.034
	Age	**−0.313**	**−0.344**	**<0.001**	**−0.443**	**−0.160**
	NART-R	**0.290**	**0.212**	**0.001**	**0.125**	**0.455**
	Sex	2.541	0.123	0.054	−0.358	5.797
	Depression	−0.141	−0.072	0.415	−0.476	0.165
	Anxiety	0.118	0.055	0.525	−0.231	0.477
8	(Constant)	**71.879**		**<0.001**	**50.845**	**90.715**
	BE4PTA	−0.024	−0.050	0.651	−0.117	0.058
	BE2PTA	−0.016	−0.046	0.678	−0.095	0.072
	4PTAsym	−0.003	−0.006	0.953	−0.109	0.104
	2PTAsym	−0.074	−0.128	0.244	−0.189	0.034
	Age	**−0.326**	**−0.358**	**<0.001**	**−0.458**	**−0.164**
	NART-R	**0.300**	**0.218**	**0.001**	**0.132**	**0.464**
	Sex	**2.613**	**0.127**	**0.049**	−0.293	5.896
	Depression	−0.094	−0.048	0.596	−0.427	0.218
	Anxiety	0.161	0.075	0.421	−0.226	0.553
	Stress	−0.104	−0.064	0.414	−0.362	0.154

BE4PTA, Speech Better-Ear Pure-Tone Average; BE2PTA, High-Frequency Better-Ear Pure-Tone Average; 4PTAsym, Speech-Frequency Hearing-Loss Asymmetry; 2PTAsym, High-Frequency Hearing-Loss Asymmetry; NART-R, Revised National Adult Reading Test. ^a^Unless otherwise noted, bootstrap results are based on 2,000 wild bootstrap samples. Bold values indicate statistically significant effects.

**Table 5:** Wild bootstrapped model estimates for steps 1 to 8 in hierarchical regression analyses of whether better-ear speech-frequency hearing (BE4PTA) and better-ear high-frequency hearing (BE2PTA) predict performance on the paired-associates-learning task, controlling for the covariates of speech-frequency-hearing asymmetry, high-frequency-hearing asymmetry, age, premorbid intelligence (NART-R), sex, depression, anxiety, and stress (*N* = 241).

Model		*B*	*β*	Sig. (two-tailed)	BCa 95% Confidence interval^a^	
					Lower	Upper
1	(Constant)	**−26.275**		**<0.001**	**−32.443**	**−20.240**
	BE4PTA	0.206	0.137	0.185	−0.108	0.533
	BE2PTA	**−0.407**	**−0.368**	**0.001**	**−0.631**	**−0.186**
2	(Constant)	**−26.368**		**<0.001**	**−33.662**	**−19.001**
	BE4PTA	0.242	0.161	0.129	−0.080	0.567
	BE2PTA	**−0.460**	**−0.415**	**<0.001**	**−0.680**	**−0.239**
	4PTAsym	0.316	0.197	0.051	−0.013	0.637
	2PTAsym	−0.169	−0.093	0.377	−0.536	0.204
3	(Constant)	**34.151**		**0.002**	**9.362**	**55.525**
	BE4PTA	0.002	0.001	0.989	−0.345	0.354
	BE2PTA	−0.210	−0.190	0.137	−0.455	0.038
	4PTAsym	0.253	0.158	0.114	−0.067	0.582
	2PTAsym	−0.158	−0.087	0.406	−0.534	0.216
	Age	**−0.994**	**−0.345**	**<0.001**	**−1.270**	**−0.673**
4	(Constant)	−34.273		0.313	−105.516	33.814
	BE4PTA	0.065	0.043	0.704	−0.282	0.413
	BE2PTA	−0.199	−0.179	0.165	−0.442	0.046
	4PTAsym	0.261	0.163	0.108	−0.055	0.590
	2PTAsym	−0.169	−0.093	0.380	−0.543	0.206
	Age	**−1.075**	**−0.373**	**<0.001**	**−1.368**	**−0.735**
	NART-R	**0.634**	**0.146**	**0.030**	**0.090**	**1.198**
5	(Constant)	−38.144		0.258	−112.735	31.467
	BE4PTA	0.056	0.037	0.746	−0.295	0.413
	BE2PTA	−0.188	−0.170	0.184	−0.430	0.062
	4PTAsym	0.261	0.163	0.107	−0.055	0.590
	2PTAsym	−0.172	−0.094	0.373	−0.544	0.202
	Age	**−1.050**	**−0.365**	**<0.001**	**−1.341**	**−0.703**
	NART-R	**0.617**	**0.142**	**0.038**	**0.050**	**1.194**
	Sex	2.670	0.041	0.488	−3.413	10.152
6	(Constant)	−38.748		0.253	−112.982	30.431
	BE4PTA	0.061	0.041	0.718	−0.287	0.412
	BE2PTA	−0.185	−0.167	0.186	−0.424	0.063
	4PTAsym	0.279	0.174	0.107	−0.038	0.622
	2PTAsym	−0.183	−0.100	0.348	−0.575	0.202
	Age	**−1.051**	**−0.365**	**<0.001**	**−1.343**	**−0.695**
	NART-R	**0.625**	**0.144**	**0.036**	**0.073**	**1.208**
	Sex	2.705	0.041	0.484	−3.386	10.214
	Depression	−0.159	−0.026	0.731	−0.985	0.609
7	(Constant)	−38.760		0.255	−112.946	30.344
	BE4PTA	0.058	0.039	0.722	−0.304	0.417
	BE2PTA	−0.182	−0.165	0.210	−0.424	0.066
	4PTAsym	0.279	0.174	0.106	−0.033	0.618
	2PTAsym	−0.187	−0.103	0.344	−0.593	0.217
	Age	**−1.052**	**−0.366**	**<0.001**	**−1.346**	**−0.706**
	NART-R	**0.625**	**0.144**	**0.037**	**0.073**	**1.209**
	Sex	2.718	0.042	0.486	−3.438	10.262
	Depression	−0.202	−0.033	0.712	−1.380	0.873
	Anxiety	0.075	0.011	0.908	−1.003	1.210
8	(Constant)	−38.633		0.254	−112.496	30.069
	BE4PTA	0.057	0.038	0.730	−0.309	0.416
	BE2PTA	−0.179	−0.162	0.226	−0.428	0.078
	4PTAsym	0.280	0.175	0.105	−0.037	0.620
	2PTAsym	−0.187	−0.103	0.345	−0.593	0.217
	Age	**−1.069**	**−0.371**	**<0.001**	**−1.393**	**−0.694**
	NART-R	**0.637**	**0.147**	**0.039**	**0.056**	**1.217**
	Sex	2.808	0.043	0.465	−3.266	10.201
	Depression	−0.143	−0.023	0.814	−1.300	0.984
	Anxiety	0.128	0.019	0.860	−1.023	1.346
	Stress	−0.130	−0.025	0.759	−0.881	0.643

BE4PTA, Speech Better-Ear Pure-Tone Average; BE2PTA, High-Frequency Better-Ear Pure-Tone Average; 4PTAsym, Speech-Frequency Hearing-Loss Asymmetry; 2PTAsym, High-Frequency Hearing-Loss Asymmetry; NART-R, Revised National Adult Reading Test. ^a^Unless otherwise noted, bootstrap results are based on 2,000 wild bootstrap samples. Bold values indicate statistically significant effects.

**Table 6:** Wild bootstrapped model estimates for steps 1 to 8 in hierarchical regression analyses of whether better-ear speech-frequency hearing (BE4PTA) and better-ear high-frequency hearing (BE2PTA) predict total errors on the spatial-working-memory task, controlling for the covariates of speech-frequency-hearing asymmetry, high-frequency-hearing asymmetry, age, premorbid intelligence (NART-R), sex, depression, anxiety, and stress (*N* = 241).

Model		*B*	*β*	Sig. (two-tailed)	BCa 95% Confidence interval^a^	
					Lower	Upper
1	(Constant)	**−24.747**		**<0.001**	**−28.944**	**−20.615**
	BE4PTA	−0.145	−0.140	0.142	−0.304	0.036
	BE2PTA	−0.105	−0.138	0.142	−0.286	0.045
2	(Constant)	**−24.414**		**<0.001**	**−29.231**	**−19.513**
	BE4PTA	−0.122	−0.117	0.223	−0.279	0.057
	BE2PTA	−0.144	−0.189	0.071	−0.338	0.017
	4PTAsym	**0.232**	**0.210**	**0.032**	**0.029**	**0.447**
	2PTAsym	−0.147	−0.117	0.234	−0.379	0.090
3	(Constant)	**35.402**		**<0.001**	**22.796**	**48.276**
	BE4PTA	**−0.359**	**−0.346**	**<0.001**	**−0.543**	**−0.166**
	BE2PTA	0.103	0.134	0.207	−0.072	0.263
	4PTAsym	0.170	0.154	0.118	−0.029	0.380
	2PTAsym	−0.136	−0.108	0.270	−0.377	0.104
	Age	**−0.983**	**−0.495**	**<0.001**	**−1.205**	**−0.777**
4	(Constant)	−3.175		0.851	−37.180	30.210
	BE4PTA	**−0.323**	**−0.312**	**0.001**	**−0.509**	**−0.115**
	BE2PTA	0.109	0.143	0.182	−0.065	0.272
	4PTAsym	0.174	0.158	0.113	−0.025	0.385
	2PTAsym	−0.142	−0.113	0.254	−0.384	0.097
	Age	**−1.028**	**−0.518**	**<0.001**	**−1.254**	**−0.814**
	NART-R	**0.358**	**0.119**	**0.023**	**0.064**	**0.661**
5	(Constant)	3.215		0.866	−32.006	37.880
	BE4PTA	**−0.308**	**−0.297**	**0.003**	**−0.496**	**−0.098**
	BE2PTA	0.092	0.120	0.267	−0.076	0.256
	4PTAsym	0.174	0.158	0.113	−0.025	0.385
	2PTAsym	−0.139	−0.110	0.263	−0.381	0.101
	Age	**−1.068**	**−0.538**	**<0.001**	**−1.292**	**−0.863**
	NART-R	**0.386**	**0.129**	**0.014**	**0.091**	**0.680**
	Sex	−4.408	−0.098	0.058	−8.693	0.140
6	(Constant)	3.535		0.857	−32.090	38.185
	BE4PTA	**−0.311**	**−0.300**	**0.002**	**−0.504**	**−0.097**
	BE2PTA	0.090	0.118	0.276	−0.079	0.252
	4PTAsym	0.165	0.149	0.174	−0.056	0.406
	2PTAsym	−0.133	−0.105	0.312	−0.383	0.111
	Age	**−1.068**	**−0.538**	**<0.001**	**−1.291**	**−0.864**
	NART-R	**0.382**	**0.127**	**0.016**	**0.086**	**0.677**
	Sex	−4.426	−0.098	0.058	−8.707	0.148
	Depression	0.084	0.020	0.717	−0.346	0.521
7	(Constant)	3.586		0.855	−32.042	38.240
	BE4PTA	**−0.299**	**−0.289**	**0.004**	**−0.495**	**−0.090**
	BE2PTA	0.081	0.106	0.324	−0.086	0.240
	4PTAsym	0.163	0.147	0.180	−0.056	0.403
	2PTAsym	−0.116	−0.092	0.389	−0.375	0.138
	Age	**−1.061**	**−0.535**	**<0.001**	**−1.284**	**−0.857**
	NART-R	**0.382**	**0.128**	**0.016**	**0.087**	**0.676**
	Sex	−4.481	−0.100	0.056	−8.820	0.105
	Depression	0.266	0.062	0.443	−0.374	0.899
	Anxiety	−0.317	−0.068	0.413	−1.064	0.412
8	(Constant)	3.762		0.845	−31.699	38.322
	BE4PTA	**−0.301**	**−0.290**	**0.005**	**−0.498**	**−0.091**
	BE2PTA	0.085	0.111	0.305	−0.081	0.245
	4PTAsym	0.164	0.149	0.179	−0.053	0.405
	2PTAsym	−0.117	−0.093	0.391	−0.376	0.137
	Age	**−1.084**	**−0.546**	**<0.001**	**−1.315**	**−0.857**
	NART-R	**0.398**	**0.133**	**0.018**	**0.086**	**0.710**
	Sex	−4.356	−0.097	0.067	−8.584	0.181
	Depression	0.348	0.082	0.322	−0.296	0.967
	Anxiety	−0.244	−0.052	0.562	−1.005	0.521
	Stress	−0.180	−0.051	0.487	−0.707	0.317

BE4PTA, Speech Better-Ear Pure-Tone Average; BE2PTA, High-Frequency Better-Ear Pure-Tone Average; 4PTAsym, Speech-Frequency Hearing-Loss Asymmetry; 2PTAsym, High-Frequency Hearing-Loss Asymmetry; NART-R, Revised National Adult Reading Test. ^a^Unless otherwise noted, bootstrap results are based on 2,000 wild bootstrap samples. Bold values indicate statistically significant effects.

**Table 7:** Wild bootstrapped model estimates for steps 1 to 8 in hierarchical regression analyses of whether better-ear speech-frequency hearing (BE4PTA) and better-ear high-frequency hearing (BE2PTA) predict strategy scores on the spatial-working-memory task, controlling for the covariates of speech-frequency-hearing asymmetry, high-frequency-hearing asymmetry, age, premorbid intelligence (NART-R), sex, depression, anxiety, and stress (*N* = 241).

Model		*B*	*β*	Sig. (two-tailed)	BCa 95% Confidence interval^a^	
					Lower	Upper
1	(Constant)	**−31.928**		**<0.001**	**−33.145**	**−30.671**
	BE4PTA	**−0.083**	**−0.303**	**0.009**	**−0.137**	**−0.026**
	BE2PTA	0.015	0.077	0.502	−0.033	0.064
2	(Constant)	**−31.921**		**<0.001**	**−33.372**	**−30.417**
	BE4PTA	**−0.081**	**−0.296**	**0.010**	**−0.136**	**−0.023**
	BE2PTA	0.012	0.062	0.601	−0.038	0.061
	4PTAsym	0.018	0.062	0.551	−0.037	0.075
	2PTAsym	−0.010	−0.031	0.765	−0.077	0.054
3	(Constant)	**−20.183**		**<0.001**	**−23.709**	**−16.398**
	BE4PTA	**−0.127**	**−0.467**	**<0.001**	**−0.190**	**−0.067**
	BE2PTA	**0.061**	**0.303**	**0.014**	**0.012**	**0.110**
	4PTAsym	0.006	0.021	0.843	−0.049	0.065
	2PTAsym	−0.008	−0.025	0.807	−0.076	0.056
	Age	**−0.193**	**−0.369**	**<0.001**	**−0.259**	**−0.133**
4	(Constant)	**−24.636**		**<0.001**	**−34.819**	**−14.880**
	BE4PTA	**−0.123**	**−0.451**	**<0.001**	**−0.185**	**−0.060**
	BE2PTA	**0.061**	**0.306**	**0.012**	**0.013**	**0.110**
	4PTAsym	0.007	0.022	0.827	−0.048	0.064
	2PTAsym	−0.009	−0.027	0.791	−0.078	0.055
	Age	**−0.198**	**−0.379**	**<0.001**	**−0.266**	**−0.136**
	NART-R	0.041	0.052	0.365	−0.040	0.131
5	(Constant)	**−21.302**		**<0.001**	**−32.347**	**−10.820**
	BE4PTA	**−0.115**	**−0.423**	**<0.001**	**−0.180**	**−0.052**
	BE2PTA	**0.052**	**0.261**	**0.034**	**0.004**	**0.102**
	4PTAsym	0.006	0.022	0.832	−0.048	0.064
	2PTAsym	−0.007	−0.022	0.834	−0.077	0.058
	Age	**−0.219**	**−0.419**	**<0.001**	**−0.286**	**−0.157**
	NART	0.056	0.072	0.216	−0.023	0.144
	Sex	**−2.300**	**−0.194**	**0.002**	**−3.640**	**−0.967**
6	(Constant)	**−20.991**		**<0.001**	**−31.926**	**−10.574**
	BE4PTA	**−0.118**	**−0.432**	**<0.001**	**−0.182**	**−0.055**
	BE2PTA	**0.051**	**0.252**	**0.042**	**0.003**	**0.100**
	4PTAsym	−0.003	−0.009	0.933	−0.056	0.056
	2PTAsym	−0.001	−0.003	0.974	−0.072	0.065
	Age	−0.219	−0.419	<0.001	−0.284	−0.157
	NART-R	0.052	0.066	0.258	−0.028	0.138
	Sex	**−2.318**	**−0.196**	**0.002**	**−3.650**	**−1.003**
	Depression	0.082	0.073	0.170	−0.041	0.197
7	(Constant)	**−20.992**		**<0.001**	**−31.945**	**−10.571**
	BE4PTA	**−0.118**	**−0.433**	**<0.001**	**−0.182**	**−0.055**
	BE2PTA	**0.051**	**0.253**	**0.047**	**0.002**	**0.101**
	4PTAsym	−0.002	−0.009	0.934	−0.056	0.055
	2PTAsym	−0.001	−0.004	0.965	−0.073	0.067
	Age	**−0.219**	**−0.419**	**<0.001**	**−0.284**	**−0.157**
	NART-R	0.052	0.066	0.260	−0.027	0.138
	Sex	**−2.316**	**−0.196**	**0.002**	**−3.647**	**−0.995**
	Depression	0.077	0.069	0.312	−0.081	0.221
	Anxiety	0.008	0.006	0.943	−0.184	0.196
8	(Constant)	**−20.965**		**<0.001**	**−31.865**	**−10.485**
	BE4PTA	**−0.118**	**−0.434**	**<0.001**	**−0.181**	**−0.056**
	BE2PTA	**0.051**	**0.257**	**0.045**	**0.002**	**0.102**
	4PTAsym	−0.002	−0.008	0.940	−0.056	0.056
	2PTAsym	−0.002	−0.005	0.965	−0.073	0.067
	Age	**−0.222**	**−0.426**	**<0.001**	**−0.290**	**−0.158**
	NART-R	0.054	0.069	0.261	−0.026	0.142
	Sex	**−2.297**	**−0.194**	**0.002**	**−3.626**	**−0.957**
	Depression	0.090	0.080	0.269	−0.077	0.251
	Anxiety	0.019	0.015	0.851	−0.179	0.216
	Stress	−0.027	−0.029	0.737	−0.180	0.122

BE4PTA, Speech Better-Ear Pure-Tone Average; BE2PTA, High-Frequency Better-Ear Pure-Tone Average; 4PTAsym, Speech-Frequency Hearing-Loss Asymmetry; 2PTAsym, High-Frequency Hearing-Loss Asymmetry; NART-R, Revised National Adult Reading Test. ^a^Unless otherwise noted, bootstrap results are based on 2,000 wild bootstrap samples. Bold values indicate statistically significant effects.

However, at step 3, age rendered all abovementioned effects nonsignificant, but also introduced some new hearing-loss-related effects; namely, significant negative βs for BE4PTA in the AST, SWMTE, and SWM_strategy_ models. This result suggested that age was a suppressor variable for speech-frequency hearing loss, in turn, suggesting that, when controlling for age-related increases in high-frequency hearing loss, and cognitive decline due to both, greater speech-frequency hearing loss predicted some reduction in executive function and working memory. The addition of age also produced a significant positive β for BE2PTA in the SWM_strategy_ model, counterintuitively suggesting that greater high-frequency hearing loss produced lower strategy reliance (i.e., better performance) in the SWM task, after accounting for age effects. These results remained largely unchanged, in terms of statistical significance, when adding NART-R, sex, and mental-health estimates to the model in steps 4 to 8.

In the final (step-8) models, additional to the abovementioned effects, the covariates of age and NART-R were significant for the AST, DMS, PAL, and SWMTE models. Meanwhile, age and sex were significant for the SWM_strategy_ model_sexsexsex_. For robustness, we applied the Hochberg step-up multiplicity correction to the bootstrapped *p*-values of our step-8 models—reported in the Online Supplement (https://osf.io/pg5fm/). Following these corrections, BE4PTA became marginally non-significant in the AST model (adjusted *p* < 0.015, obtained *p* = 0.019), and NART-R became marginally non-significant in the SWMTE model (adjusted *p* < 0.015, obtained *p* = 0.018); further, sex became non-significant in the DMS model (adjusted *p* < 0.015, obtained *p* = 0.049), NART-R became non-significant in the PAL model (adjusted *p* < 0.010, obtained *p* = 0.039), and BE2PTA became non-significant in the SWM_strategy_ model (adjusted *p* < 0.020, obtained *p* = 0.045). All other effects remained significant. Adjusted *R*^2^ values for the AST, DMS, PAL, SWMTE, and SWM_strategy_ models were 0.26, 0.20, 0.18, 0.30, and 0.19, respectively.

## 4 Discussion

In the current study, we aimed to investigate whether high-frequency hearing loss impacts cognitive function in older adults beyond the impact of speech-frequency hearing loss. It was firstly hypothesised that greater speech and high-frequency hearing loss would be associated with lower cognitive ability, which the current study found support for. It was further hypothesised that greater high-frequency hearing loss would predict lower cognitive functioning beyond the influence of speech-frequency hearing loss, which was largely unsupported.

First, we found that adult’s better-ear, pure-tone averages in the speech (BE4PTA) and high-frequency (BE2PTA) ranges correlated significantly with non-verbal versions of the attention-switching (AST), delayed matching to sample (DMS), paired associates learning (PAL), and spatial working memory (SWM) tasks of the Cambridge neuropsychological test automated battery (CANTAB). That is, greater speech and high-frequency hearing loss was associated with poorer cognitive flexibility (i.e., AST), short-term-memory capacity (i.e., DMS), episodic memory and learning (i.e., PAL), and working-memory ability (i.e., SWM); this generally suggested hearing loss to be associated with worse executive function and memory capacity. While BE2PTA initially showed a unique effect over BE4PTA in the DMS and PAL regression models, age later accounted for these effects; at the final step of both models, only age and premorbid IQ (NART-R) demonstrated significant beta-weights. Consequently, although high-frequency hearing loss accounted for variance in visual learning and short-term memory capacity beyond speech-frequency hearing loss, this unique variance was accounted for by age-related changes in cognition and hearing.

Conversely, in AST and SWM regression models, neither BE4PTA nor BE2PTA initially demonstrated significant beta-weights. However, adding age to these models produced significant beta-weights for both BE4PTA and BE2PTA, suggesting age was a suppressor variable in these cases (for info on suppressor effects, see Conger, 1974). Regarding the suppressed BE4PTA effects first, greater speech-frequency hearing loss was associated with worse cognitive flexibility (i.e., AST) and working-memory (i.e., SWM errors & strategy) performance. Thus, when controlling for high-frequency hearing loss and age, speech-frequency hearing loss had a unique effect on aspects of cognition related to executive function. Most immediately, this finding expands upon those of Jayakody et al. (2018), who found that speech-frequency hearing loss uniquely predicted (controlling for age) performance on memory and executive-function tasks from the same test battery. Furthermore, the present finding adds to previous evidence showing a negative effect of hearing loss on executive function (Taljaard et al., 2016; Alattar et al., 2019; Brewster et al., 2020; Lin et al., 2013; Loughrey et al., 2018; Wild et al., 2012), and suggests that it is due uniquely to speech-frequency hearing loss. Accordingly, the focus on speech-range hearing frequencies in previous cognition-focused work may be justified. However, it should be noted that applying Hochberg familywise-error correction to AST-model results caused the effect of BE4PTA to become marginally non-significant (adjusted *p* < 0.015, obtained *p* = 0.019), though the SWMTE-model effect remained significant. Furthermore, speech-frequency hearing loss did not significantly correlate with age in the current study, unlike high-frequency hearing loss; this could potentially be due to the inclusion of participants with non-age-related hearing losses, which could have produced more-severe speech-frequency hearing loss in younger participants, thus weakening the expected correlation. Therefore, future studies with large sample sizes, a longitudinal design, and a more-diverse range of control variables will be required to provide stronger evidence for a unique effect of speech-frequency hearing loss on cognitive ability.

As for the suppressed BE2PTA effect, less high-frequency hearing loss was associated with greater reliance on rote strategies in the SWM task (i.e., worse performance), going against the deleterious effect typically found between hearing loss and cognition (for review, see Livingston et al., 2020). A possible, though speculative, explanation for this finding, could be that those with better high-frequency hearing found cognitive testing generally easier; this may then have caused lower attentiveness via greater boredom (Hunter and Eastwood, 2018), possibly promoting greater use of rote strategies to complete tasks. This speculation could also be supported by the fact that the SWM-strategy model alone showed no unique effect of premorbid IQ (NART-R), suggesting that strategy-use was not related to cognitive ability; thus, other aspects of performance such as motivation could have played a role. However, this speculation and the associated effect would need to be tested in future work; this is further encouraged by the fact that the Hochberg multiplicity corrections we applied to our models rendered the suppressed BE2PTA effect non-significant.

The present results firstly provide further evidence that hearing loss has a negative association with cognition (Livingston et al., 2020; Lin et al., 2011; Loughrey et al., 2018), further encouraging investigations on whether hearing-loss intervention (e.g., hearing aids, cochlear implants) can reduce cognitive decline (Lin et al., 2023; Jayakody et al., 2020; Sarant et al., 2019; Sarant et al., 2020). Furthermore, our results suggest that the detrimental cognitive effects of high-frequency, but not speech-frequency, hearing loss are largely related to ageing. In contrast, previous work on the cognitive effects of high-frequency hearing loss has found evidence for an effect with Chinese-speaking older adults (aged 60+; Diao et al., 2021; Fu et al., 2023), Belgian younger-to-older adults (aged 16 to 69 years), and Swedish students (aged 23–66; Waechter et al., 2022); though, the first did not control for age, and the second mixed speech- and high-frequency hearing into a single composite measure. Notably, Waechter et al. (2022) measured extended high-frequency hearing (10, 12.5, 14, 16 kHz), while the current study used 6 and 8 kHz. It may, then, be possible that non-age-related cognitive effects of hearing loss are more reliably observed in the extended high-frequency ranges; however, further research will be needed to ascertain this, specifically using comprehensive cognitive testing alongside expanded hearing-frequency measures. It is also possible that calculating a high-frequency PTA from only two estimates (i.e., 6 & 8 kHz) in the current study provided a less-reliable average, perhaps suggesting that the present results be interpreted with caution. Overall, it is currently unclear whether high-frequency hearing loss contributes to cognitive decline beyond the effects of ageing and speech-frequency hearing loss.

The current results could also provide some limited insight into discussions over the causal mechanisms between peripheral hearing loss and cognition. Several relevant hypotheses have been proposed. For example, Griffiths et al. (2020) discussed four hypotheses: (1) common-pathology hypothesis; (2) impoverished-environment hypothesis; (3) cognitive-load hypothesis; and (4) cognition-pathology interaction hypothesis. The common pathology hypothesis suggests that ageing-related pathology of the cochlea, auditory pathway, and cortex produce the observed cognition-hearing correlation. The current results could lend some credence to this hypothesis, given that age accounted for most of the hearing-related cognitive effects we found, particularly for the higher frequencies (6 and 8 kHz). However, as discussed above, speech-frequency hearing loss demonstrated effects on cognitive flexibility and working memory beyond ageing, perhaps suggesting that speech and language ability uniquely relate to more active aspects of cognition. Indeed, previous work has shown that the ability to discern speech in noise relates to cognition and cognitive decline (Häggström et al., 2020; Mohammed et al., 2022; Jalaei et al., 2019). Such speech- and communication-related effects could also accord with the impoverished-environment hypothesis, which generally suggests that hearing loss impacts cognition by degrading auditory information received by the brain. More specifically, the degradation in auditory information could decrement socially-relevant information and functioning, potentially leading to social withdrawal and loneliness (Shukla et al., 2020; Jayakody et al., 2022); information impoverishment could also encourage deleterious changes to brain anatomy (Neuschwander et al., 2019; Qiu et al., 2024; Wingfield and Peelle, 2015). Meanwhile, the cognitive-load hypothesis suggests that deficits caused by information impoverishment are compensated for by greater recruitment of cognitive resources, leaving fewer resources for other demanding cognitive tasks. Our results could align with this hypothesis, given that speech-frequency hearing loss only uniquely affected cognitive tasks that demanded more than simple memory storage and recall. Finally, the cognition-pathology interaction hypothesis suggests that the processes proposed by hypotheses 2 and 3 interact with neuronal changes due to dementia pathology, which the present results cannot provide further clarity on. In sum, then, our results suggest that while ageing-related changes may underly co-deficits in cognition and high-frequency hearing (common-cause hypothesis), speech-frequency hearing loss may more-directly impact higher-order cognitive abilities via information degradation (impoverished-environment hypothesis) and resultant, compensatory cognitive-load increases (cognitive-load hypothesis). However, future work will be required to make firm conclusions regarding the causal mechanisms linking hearing loss and cognition.

Regarding other noteworthy effects found in the current study, it was firstly observed that hearing loss in speech- and high-frequency ranges correlated positively with depression, anxiety, and stress symptoms. This corroborates previous findings (Zivkovic Marinkov et al., 2022, for reviews, see Bigelow et al., 2020; Blazer and Tucci, 2019; Fu et al., 2022; Jiang et al., 2020; Jayakody et al., 2018), suggesting that psychological distress is a common aspect of hearing loss; note that some of the mental-health data from Jayakody et al. (2018) is included in the current study. We further found that speech-frequency-hearing asymmetry correlated positively with depression, anxiety, and stress symptoms, while asymmetry in high-frequency hearing only correlated positively with anxiety symptoms; supplementary analyses showed that these correlations survived after controlling for hearing-loss severity in both frequency ranges (see Online Supplement) (https://osf.io/pg5fm/). This is a somewhat novel finding, and suggests that asymmetry in hearing has deleterious psychological effects, regardless of hearing-loss severity. A similar finding from a recent study (Olze et al., 2023) found that subjective hearing quality correlated negatively with anxiety and depressive symptoms in an asymmetrical-hearing-loss, but not a bilateral-hearing-loss, group. Negative effects of unilateral hearing loss (i.e., hearing loss in one ear only) on psychological functioning have also been found in a sample of young Korean men (Song et al., 2021); while unilateral hearing loss can be distinct in terms of aetiology and presentation, we simply wish to emphasise that asymmetry generally appears to carry psychological effects. One possible reason for these findings could be that people with asymmetrical hearing loss are more aware of hearing loss in their worse ear, given its comparatively poorer performance to the better ear; this increased awareness could then contribute to increased psychological distress, which could be further fuelled by a fear of losing hearing in the better ear (Lucas et al., 2018). Furthermore, many post-lingual asymmetrical hearing losses are sudden (Usami et al., 2017), which could produce more distress than a more gradual loss. However, future research is needed to understand by what mechanisms hearing asymmetry affects mental health. Finally, depression, anxiety, and stress scores were not found to correlate with any cognitive measures in the present study, somewhat contradicting previous research (Chavez-Baldini et al., 2023; Iosifescu, 2012; Millan et al., 2012). However, this finding should be interpreted with caution, as there was generally low mental-health symptomology in our sample, with previous research focussing on those clinically diagnosed with various psychopathologies.

### 4.1 Clinical implications

In practical terms, the current results firstly reemphasise the need for greater awareness of the comorbidity between hearing loss and cognitive impairment. Indeed, recent studies suggest that significant gaps persist in healthcare systems around the world that limit the efficacy of care for patients with comorbid hearing loss and cognitive impairment (Leroi et al., 2019; Völter et al., 2020; Littlejohn et al., 2021; Jayakody et al., 2024). Furthermore, our findings suggest that clinical audiologists should pay particular attention to the mental health of those with asymmetrical hearing loss, as we found more mental-health symptoms in those with greater hearing asymmetry, regardless of hearing-loss severity. Indeed, it may be that those with asymmetrical hearing loss require additional counselling on top of audiological interventions, in order to cope with their condition. Finally, while we found no direct connection between high-frequency hearing loss and cognition, this conflicts with previous research using extended high-frequency hearing measures; therefore, there may be justification for including extended high-frequencies in audiological check-ups.

### 4.2 Limitations

As already discussed above, one possible limitation was that our measure of high-frequency hearing loss was only based on two frequencies, meaning that the mean estimate may have had lower internal-consistency reliability, thus limiting the potential correlations that could be found with other variables. Furthermore, while we conducted supplementary analyses that provided no evidence for confounding effects (see Online Supplement) (https://osf.io/pg5fm/), it is possible that the inclusion of participants with different hearing-loss aetiologies influenced our results; indeed, the lack of correlation found between speech-frequency hearing loss and age could have been due to this. We were also unable to include other control variables, such as education level, previous hearing-aid usage, and tinnitus status in our main analyses, namely due to a high degree of missing data for these variables; however, supplementary analyses suggested that their inclusion in main analyses would not have influenced results (see Online Supplement) (https://osf.io/pg5fm/). Moreover, despite the current study’s larger sample size, its cross-sectional design limits the drawable conclusions, as the degree of transient error is unknown. Therefore, future studies should seek to reproduce the present results with a longitudinal design, while also correcting for the other limitations noted above.

## 5 Conclusion

In summary, both speech and high-frequency hearing loss are significantly associated with cognition and mental health; however, only speech-frequency hearing loss showed evidence of cognitive impacts beyond the effects of ageing. Despite this, previous findings suggest that cognition is impacted by hearing loss in higher frequencies, especially those in the extended range that were not presently investigated. Thus, future investigations are needed to clarify high-frequency hearing loss’ impact on cognitive decline, which could help to improve outcomes for the millions of older adults afflicted by both conditions.

## Data Availability

The datasets presented in this study can be found in online repositories. The names of the repository/repositories and accession number(s) can be found in the article/supplementary material.
